# Early Severe Cortical Involvement and Novel *FUCA1* Mutations in a Pediatric Fucosidosis Case

**DOI:** 10.1002/mgg3.70070

**Published:** 2025-01-25

**Authors:** Mar Jiménez de la Peña, Sara López‐Martín, Daniel Martín Fernández‐Mayoralas, Ana Laura Fernández‐Perrone, Ana Jiménez de Domingo, Pilar Tirado, Beatriz Calleja‐Pérez, Sara Álvarez, Jacobo Albert, Alberto Fernández‐Jaén

**Affiliations:** ^1^ Neuroimaging Hospital Universitario Quirónsalud Madrid Spain; ^2^ Faculty of Psychology Universidad Autónoma de Madrid Madrid Spain; ^3^ Neuromottiva Madrid Spain; ^4^ Department of Pediatric Neurology Hospital Universitario Quirónsalud Madrid Spain; ^5^ Department of Pediatric Neurology Hospital Universitario La Paz Madrid Spain; ^6^ Pediatric Primary Care, C. S. Doctor Cirajas Madrid Spain; ^7^ Genomics and Medicine NIMGenetics Madrid Spain; ^8^ School of Medicine Universidad Europea de Madrid Madrid Spain

**Keywords:** cortical thickness, *FUCA*1 gene, fucosidosis, neuroimaging

## Abstract

**Background:**

Biallelic pathogenic variants in the *FUCA1* gene are associated with fucosidosis. This report describes a 4‐year‐old boy presenting with psychomotor regression, spasticity, and dystonic postures.

**Methods and Results:**

Trio‐based whole exome sequencing revealed two previously unreported loss‐of‐function variants in the *FUCA1* gene. Brain magnetic resonance imaging (MRI) findings included corpus callosum hypoplasia, white matter hypomyelination, and alterations in the globus pallidi, alongside markedly reduced cortical thickness.

**Conclusions:**

These findings suggest that cortical atrophy may occur in the early stages of fucosidosis. Early diagnosis is imperative for genetic counseling, timely investigations, and initiating early therapeutic interventions to potentially mitigate more extensive brain involvement.

## Graphical abstract

We report a 4‐year‐old boy with fucosidosis with novel pathogenic FUCA1 variants and neuroanatomical correlates. Results observed contribute to a better understanding of the genetics and pathophysiology of this rare lysosomal storage disorder.

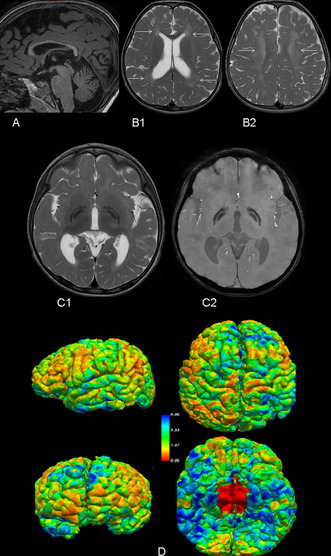

## 1 Introduction

Fucosidosis (OMIM #230000) is a rare lysosomal storage disorder caused by a deficiency of the enzyme α‐L‐fucosidase, leading to the accumulation of fucose‐containing glycolipids, glycoproteins, and oligosaccharides in various tissues. It is caused by pathogenic variants in the *FUCA1* (alpha‐L‐fucosidase 1; *612280) gene, resulting in insufficient activity of the α‐L‐fucosidase enzyme in all tissues (Saleh‐Gohari, Saeidi, and Zeighaminejad 2018; Stepien, Ciara, and Jezela‐Stanek 2020). This enzyme deficiency impairs the degradation of fucosylated compounds, leading to their accumulation in the lysosomes.

To date, 35 pathogenic variants of the *FUCA1* gene have been reported in HGMD (https://www.hgmd.cf.ac.uk/), along with 54 pathogenic or likely pathogenic variants in ClinVar (https://www.ncbi.nlm.nih.gov/clinvar/), with some overlap between the two databases. Additionally, 13 deletions and insertions have been documented. Most pathogenic variants in the *FUCA1 gene* are nonsense or missense variants, with several small deletions/insertions and splice site variations also reported.

Fucosidosis is characterized by a broad and continuous clinical spectrum without any genetic evidence for real clinical heterogeneity (Saleh‐Gohari, Saeidi, and Zeighaminejad 2018; Stepien, Ciara, and Jezela‐Stanek 2020; Willems et al. 1999). Two forms have been reported: a rapidly progressive course leading to death in infancy (type I) and a slightly milder variant, culminating in death in adulthood (type II) (Willems et al. 1999). However, subsequent studies indicate a spectrum of severity in fucosidosis (Ben Turkia et al. 2008; Willems et al. 1999).

This autosomal recessive disorder predominantly affects the central nervous system and results in progressive neurodegeneration. Neurological symptoms are pronounced in fucosidosis, with most patients presenting with global developmental delay, intellectual disability, and regression of acquired skills. Seizures are also prevalent.

Characteristic physical features of the disease include coarse facial features, hirsutism, and joint stiffness. Beyond these neurological and physical manifestations, fucosidosis can lead to systemic complications (Ben Turkia et al. 2008; Stepien, Ciara, and Jezela‐Stanek 2020; Willems et al. 1991). Commonly observed are hepatomegaly, splenomegaly, and recurrent respiratory infections, alongside skeletal abnormalities, like dysostosis multiplex and joint contractures.

Neuroimaging studies in fucosidosis have identified characteristic patterns. Magnetic resonance imaging (MRI) and magnetic resonance spectroscopy (MRS) findings have shown diffuse white‐matter hyperintensity, pallidal curvilinear streak hyperintensity, and hypomyelination in affected individuals (Mamourian et al. 2010; Oner et al. 2007; Prietsch et al. 2008). Other characteristic features include thalamic density changes and abnormalities involving the internal medullary lamina, internal capsule, and globus pallidi (Terespolsky, Clarke, and Blaser 1996). Some patients also exhibit “eye‐of‐the‐tiger” sign‐like findings, typically associated with NBIA (neurodegeneration with brain iron accumulation) (Ranganath and Patil 2023). These neuroimaging abnormalities are crucial for diagnosis and contribute to our understanding of the clinical and neuroradiological spectrum of fucosidosis (Prietsch et al. 2008), although definitive diagnosis is confirmed by enzyme activity measurement and identification of pathogenic biallelic *FUCA1* variants.

In this report, we present a 4‐year‐old Spanish boy with fucosidosis, novel pathogenic *FUCA1* variants, and unique neuroimaging findings.

## 2 Clinical Report: Methods and Results

A 4‐year‐old male, born to non‐consanguineous Spanish parents, was under evaluation for significant developmental delay and regression. His birth measurements were 3.3 kg in weight (50th percentile), 52 cm in length (50th percentile), and a cranial circumference of 31 cm (25th percentile). Developmentally, he sat at 9 months, crawled at 15 months, and achieved supported standing at 18 months, with a notable verbal regression at 14 months.

At 4 years of age, when he was evaluated in our department, the patient exhibited low trunk tone, distal hypertonia, exaggerated osteotendinous reflexes, flexor plantar reflex, and spasticity in the limbs, particularly in the lower extremities, accompanied by dyskinetic‐dystonic postures in the upper limbs. Cranial nerve examination was normal, except for a marked hearing impairment. His physical measurements were 19.5 kg (95th percentile), 105 cm (75th percentile), and 54 cm in cranial circumference (95th percentile). While he exhibited mild hypertelorism and thick lips, these features were not particularly striking or suggestive of a syndromic appearance.

At the age of three, a brain MRI revealed unspecific white matter alteration and bilateral globus pallidi involvement, with the “eye of the tiger” sign.

**FIGURE 1:**
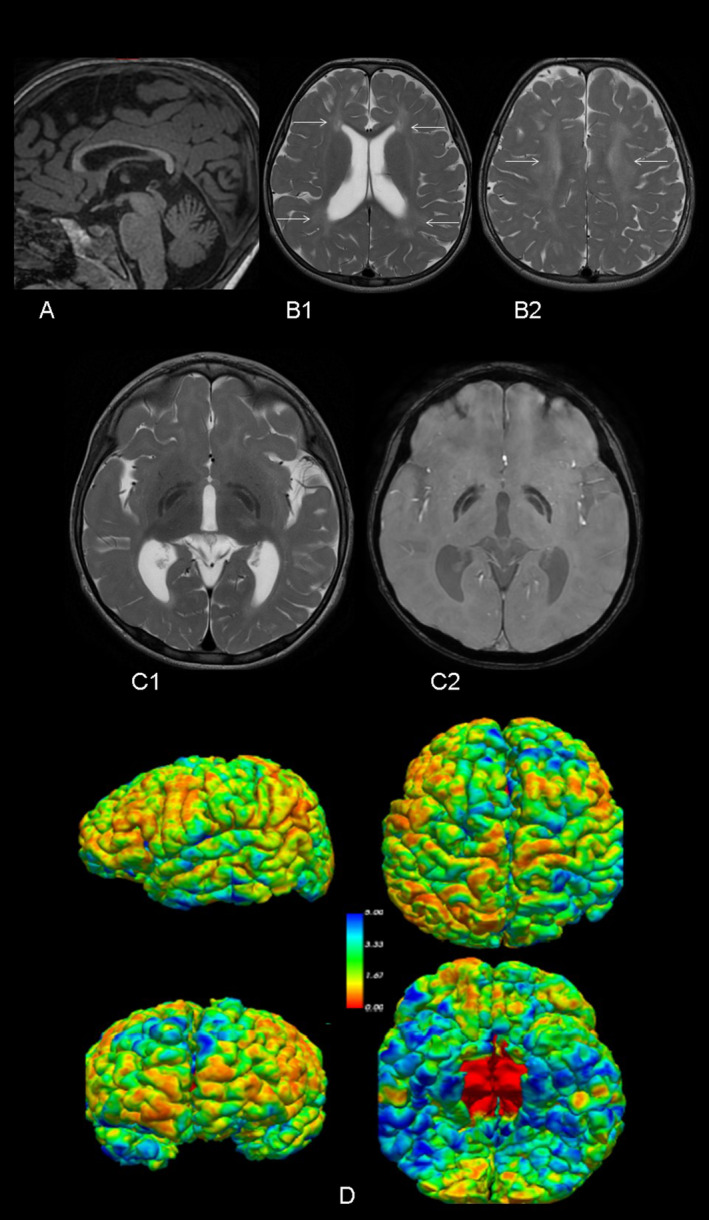
(A) T1‐weighted MPRAGE sagittal reformation showing hypoplasia of the corpus callosum; (B1, B2) T2‐weighted axial images. Symmetric hyperintensity of the corona radiata and periventricular white matter (white arrows) secondary to hypomielination; Symmetrical bilateral hypointensity of the lateral and medial portions of the globus pallidi, visible on T2‐weighted (C1) and susceptibility weighted (C2) images; (D) volumetric view of brain cortical thickness: Severely decreased cortical thickness mainly in frontal an parietal cortices.

**FIGURE 2:**
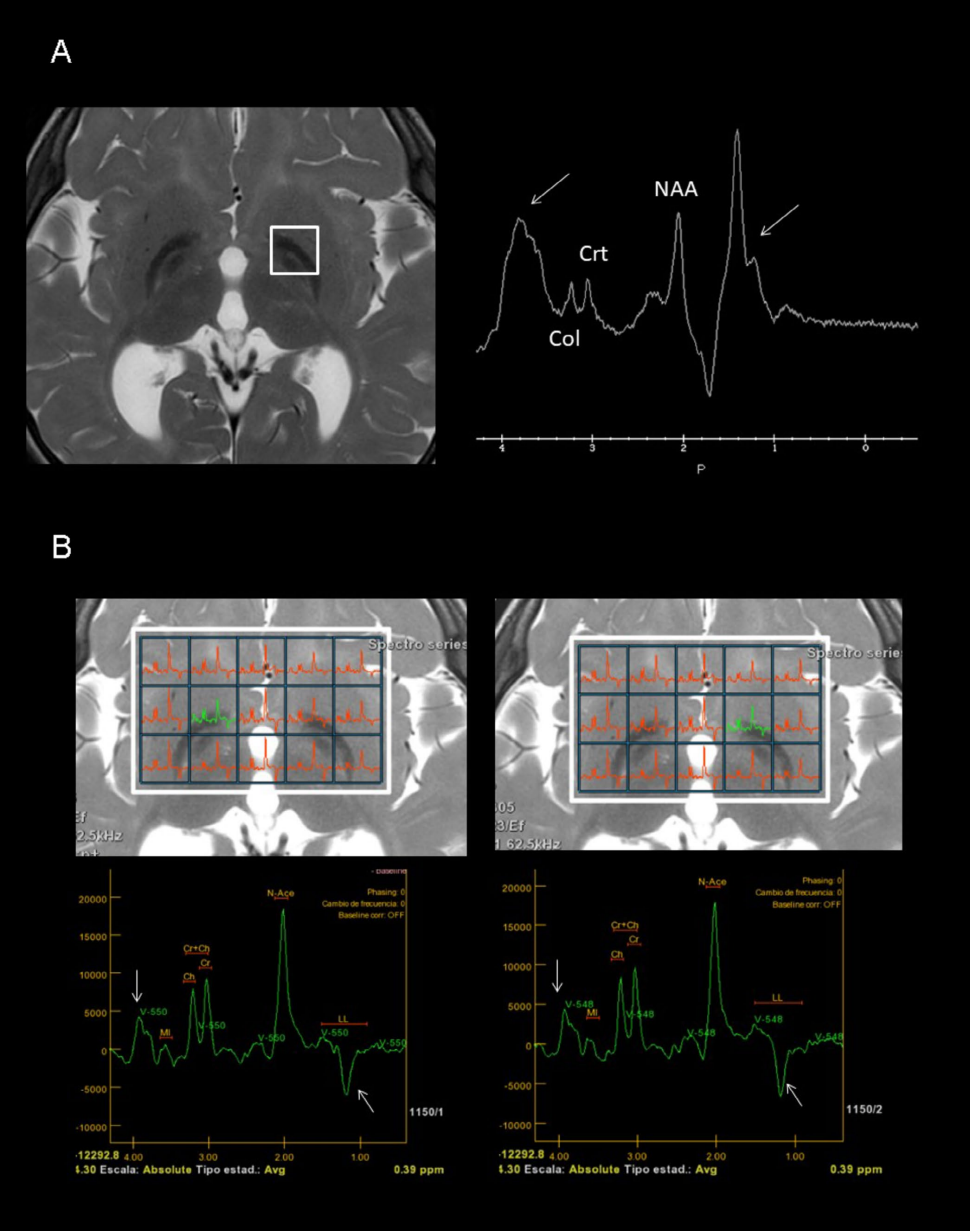
(A) Short TE single voxel 1H MR‐spectroscopy of the basal ganglia reveals a double high and broad peaks at 3.9 and 1.2 ppm. (B) Long TE multivoxel 1H MR‐spectroscopy of the basal ganglia confirms the bilateral reverse of the 1.2 ppm peak.

At the age of three, initial genetic evaluations, including karyotype, Fragile X chromosome testing, 850 k array‐CGH, and targeted exome sequencing for NBIA, yielded normal results. Further genetic analysis was pursued through whole exome sequencing (WES) in trio. Variant prioritization was performed using software developed in our laboratory. This software prioritizes undescribed loss‐of‐function (LoF) variants or previously reported pathogenic variants, filtering them by population frequency and zygosity. Additionally, it considers variants annotated in ClinVar with uncertain significance or conflicting interpretations. Most of our studies are performed on trios, allowing the inclusion of segregation‐based filters. Thus, this study identified two pathogenic variants in the *FUCA1* gene: NM_000147.4:c.856C > T; p.(Gln286*) in exon 5, carried by the healthy father, and NM_000147.4:c.539_546delinsTTTAAGGAA; p.(Gly180Valfs*2) in exon 2, carried by the healthy mother. Although both variants were loss‐of‐function, computational predictions regarding the pathogenicity of the first variant (p.(Gln286*)) were inconclusive (pathogenic by BayesDel; benign by FATHMM, EIGEN, LRT, and DANN; uncertain by MutationTaster), while the second variant (Gly180Valfs*2) was predicted to be pathogenic. Subsequent to the genetic studies, a repeat brain 3T MRI and spectroscopy were conducted, along with a 24‐h urinary oligosaccharides test, fucosidase activity assay, abdominal ultrasound, cardiological evaluation, and auditory and visual potential studies. The brain MRI showed corpus callosum hypoplasia, involvement of the corona radiata and periventricular white matter, and involvement of lateral and medial portions of both globus pallidi (Figure 1A–C2). Automated and quantified analysis of cortical thickness revealed thicknesses of 2.27 and 2.53 mm in the left and right hemispheres, respectively, below the 0.5th percentile for this age according to our own and international data (Figure 1D) (Frangou et al. 2022). Spectroscopy of the ganglia revealed a double peak at 3.9 and 1.2 ppm, suggestive of carbohydrates presence, and confirmed bilateral reverse of the 1.2 ppm peak, corresponding to fucose (Figure 2). The qualitative analysis of 24‐h urinary oligosaccharides showed a pattern compatible with fucosidosis, confirmed by the absence of fucosidase activity. The abdominal ultrasound and cardiological study were normal, as were the visual evoked potentials (VEP). The auditory potentials demonstrated bilateral moderate sensorineural hearing loss.

## 3 Discussion

We describe a new case with fucosidosis, associated with two novel pathogenic variants of the *FUCA1* gene. Fucosidosis is a rare disease with a very low incidence of < 1/200,000 (Stepien, Ciara, and Jezela‐Stanek 2020). Most of the pathogenic variants related to fucosidosis are missense or nonsense; most of affected patients have compound heterozygous variants, making it difficult to establish genotype–phenotype relationships (Stepien, Ciara, and Jezela‐Stanek 2020; Willems et al. 1999).

Developmental delay is a typical feature in most cases, and more than 40%–50% of them lose their ability to sit, stand, walk alone or speak as our case showed (Willems et al. 1991). This delay is observed at a median age of 18 months. Progressive neurological regression results in spastic tetraparesis with increased deep tendon reflexes, gradually increasing spasticity, and in some cases, with the appearance of dyskinesia and dystonia (Ben Turkia et al. 2008; Stepien, Ciara, and Jezela‐Stanek 2020; Willems et al. 1991).

According to this regression, brain MRI show increasing changes too. The most significant neuroimaging findings include extensive and progressive, symmetrical alterations in signal intensity of various white matter regions; notable observations include hyperintensity in the cerebellar, cerebral, periventricular, lobar, and subcortical white matter on T2‐weighted images (Galluzzi et al. 2001; Stepien, Ciara, and Jezela‐Stanek 2020). Signal changes in the globus pallidi are also characteristic (Galluzzi et al. 2001; Stepien, Ciara, and Jezela‐Stanek 2020). The combination of marked hypointensity of the globus pallidi on T2 sequences and hyperintensity on T1‐weighted MR sequences is typical, although neuroimaging is not sufficient for diagnosis of fucosidosis. A hypointense area on T2‐weighted imaging in the globus pallidi with associated with curvilinear T2‐hyperintense areas within lentiform nuclei, describes a sign sometimes called “eye of the tiger”. This sign has been reported neurodegeneration with brain iron accumulation diseases (Svetel et al. 2021). MRS also shows a characteristic decreased N‐acetyl aspartate/choline ratio and a typical abnormal peak at 3.8–3.9 ppm and a double peak at 1.2 ppm, suggesting great carbohydrate peaks (Ediz et al. 2016; Galluzzi et al. 2001; Mamourian et al. 2010; Oner et al. 2007).

Our case highlights three of the most relevant aspects of the neuroimaging findings in fucosidosis. First, the omnipresent white matter alterations. Second, the presence of the typical bilateral globus pallidi alterations. Third, the characteristic peaks on MRS. However, we have demonstrated an intriguing finding, the presence of a very low cortical thickness in our patient. The absence of cortical thickness studies in previous studies on neuroimaging in fucosidosis might justify the novelty of our finding. Although decreased cortical thickness may be related to an abnormal prenatal cortical development, it might be reflecting an early cortical atrophy, coherent with our patient clinical features. Additionally, previous studies have demonstrated cortico‐subcortical atrophy in the advances stages of this disease (Stepien, Ciara, and Jezela‐Stanek 2020).

The treatment options for fucosidosis are currently limited to supportive therapy. It is important to note that there is currently no approved treatment for the neurological disease‐related symptoms of fucosidosis, highlighting the need for further research and development of effective therapeutic interventions (Naumchik et al. 2020; Wang et al. 2020). However, there are potential treatment modalities that have been explored and show promise, such as bone marrow transplantation, particularly if initiated before the onset of neurological symptoms (Hopwood et al. 1993; Taylor, Farrow, and Stewart 1992). Additionally, umbilical cord blood transplantation and intracisternal enzyme replacement therapy are other treatment modalities that have been investigated (Jiang et al. 2017; Kondagari et al. 2015).

Our case demonstrates the presence of early cortical and subcortical involvement. Considering the presence of affected siblings previously described and the potential benefit of the indicated measures, early recognition of this disease is essential, both for family genetic counseling and for consideration of these potential treatment modalities (Kaur et al. 2019).

## Author Contributions

**Mar Jiménez de la Peña:** investigation, writing – original draft, software, formal analysis. **Sara López‐Martín:** investigation, writing – original draft; writing – review and editing, visualization. **Daniel Martín Fernández‐Mayoralas:** investigation, writing – review and editing. **Ana Laura Fernández‐Perrone:** investigation, writing – review and editing. **Ana Jiménez de Domingo:** investigation, writing – review and editing. **Pilar Tirado:** investigation, writing – review and editing. **Beatriz Calleja‐Pérez:** investigation, writing – review and editing. **Sara Álvarez:** software, formal analysis. **Jacobo Albert** and **Alberto Fernández‐Jaén:** conceptualization, methodology, validation, formal analysis, investigation, resources, writing – original draft, writing – review and editing, visualization, supervision, project administration.

## Ethics Statement

The study was carried out in accordance with the Declaration of Helsinki of the World Medical Association and was approved by the Local Ethics Committees. Informed consent was obtained from parents, after full explanation of the procedures.

## Conflicts of Interest

The authors declare no conflicts of interest.

## Data Availability

The datasets generated during and/or analyzed during the current study are available from the corresponding author upon reasonable request.
